# Vaccine implementation factors affecting maternal tetanus immunization in low- and middle-income countries: Results of the Maternal Immunization and Antenatal Care Situational Analysis (MIACSA) project

**DOI:** 10.1016/j.vaccine.2020.05.084

**Published:** 2020-07-14

**Authors:** M.L. Giles, C. Mantel, F.M. Muñoz, A. Moran, N. Roos, N. Yusuf, T. Diaz, M. Ahun, L.M. Nic Lochlainn, E. Wootton, J. Pathirana, S. Rendell, O. Tuncalp, M. Perut, J. Hombach, S. Merten, P. Lambach

**Affiliations:** a Department of Obstetrics and Gynaecology, Monash University, Melbourne, Australia; b MMGH Consulting, Zurich, Switzerland; c Departments of Pediatrics, Section of Infectious Diseases, and Molecular Virology and Microbiology, Baylor College of Medicine, Houston TX, USA; d Department of Maternal, Newborn, Child, Adolescent Health and Ageing, Geneva, Switzerland; e Independent Consultant, McKing Consulting, Geneva, Switzerland; f Department of Immunization, Vaccines and Biologicals (IVB), World Health Organization, Geneva, Switzerland; i Medical Research Council: Respiratory and Meningeal Pathogens Research Unit and Department of Science and Technology/ National Research Foundation: Vaccine Preventable Diseases, Faculty of Health Sciences, University of the Witwatersrand, Johannesburg, South Africa; j Department of Anthropology University of Pennsylvania, Philadelphia, USA; k Department of Sexual and Reproductive Health and Research including UNDP/UNFPA/UNICEF/WHO/World Bank Special Programme of Research, Development and Research Training in Human Reproduction, WHO, Geneva, Switzerland; l Swiss Tropical and Public Health Institute and University of Basel, Basel, Switzerland

**Keywords:** Pregnancy, Tetanus, Vaccination, Maternal Immunization, Service Delivery, LMICs, MIACSA

## Abstract

**Objectives:**

To examine the characteristics of existing maternal tetanus immunization programmes for pregnant women in low- and middle-income countries (LMICs) and to identify and understand the challenges, barriers and facilitators associated with maternal vaccine service delivery that may impact the introduction and implementation of new maternal vaccines in the future.

**Design:**

A mixed methods, cross sectional study with four data collection phases including a desk review, online survey, telephone and face-to-face interviews and in country visits.

**Setting:**

LMICs.

**Results:**

The majority of countries (84/95; 88%) had a maternal tetanus immunization policy. Countries with high protection at birth (PAB) were more likely to report tetanus toxoid-containing vaccine (TTCV) coverage targets > 90%. Less than half the countries included in this study had a TTCV coverage target of > 90%. Procurement and distribution of TTCV was nearly always the responsibility of the Expanded Programme on Immunization (EPI), however planning and management of maternal immunization was often shared between EPI and Maternal, Newborn and Child Health (MNCH) programmes. Receipt of TTCV at the same time as the antenatal care visit correlated with high PAB. Most countries (81/95; 85%) had an immunization safety surveillance system in place although only 11% could differentiate an adverse event following immunization (AEFI) in pregnant and non-pregnant women.

**Conclusions:**

Recommendations arising from the MIACSA project to strengthen existing services currently delivering maternal tetanus immunization in LMICs include establishing and maintaining vaccination targets, clearly defining responsibilities and fostering collaborations between EPI and MNCH, investing in strengthening the health workforce, improving the design and use of existing record keeping for immunization, adjusting current AEFI reporting to differentiate pregnant women and endeavoring to integrate the provision of TTCV within ANC services where appropriate.

## 1 Background

Maternal immunization has emerged as a strategy to reduce the morbidity and mortality of pregnant women and their very young infants during the vulnerable first weeks of the infant’s life via transfer of maternally-derived pathogen specific antibodies via the placenta and breast milk [1], [2], [3]. Infectious diseases, particularly pneumonia and sepsis, are leading causes of death in children under five years of age in low- and middle-income countries (LMIC) [4]. Whilst significant progress has been made in recent decades to reduce mortality in children less than five years of age, there has been less progress in reducing neonatal mortality. In 2017, the global mortality rate for children under five years of age was 39 per 1,000 live births, half the worldwide rate in 1990 [5]. Deaths in the first month of life were estimated in 2017 to be 18 per 1,000 live births, down from 31 per 1,000 live births in 2000 [5]. The Sustainable Development Goals (SDGs), launched in 2015, [6] set the target 3.2 to end preventable deaths of newborns and children under five years of age by 2030 and to reduce neonatal mortality to a maximum of 12 per 1,000 live births [7]. The most common preventable deaths include preterm birth complications, birth asphyxia, acute respiratory infections and diarrhoea [4]. Ending preventable deaths can be achieved by providing immediate and exclusive breastfeeding, access to skilled health professionals for antenatal, birth and post natal care, improved access to water and sanitation and by providing immunizations. Whilst great progress has been made globally, mortality rates in Sub-Saharan Africa and South Asia remain substantial. As a result, numerous countries from these regions risk being among the 60 countries projected to miss the SDGs in 2030 [8].

Vaccination against infectious diseases provided by the Expanded Programme on Immunization (EPI) has played a key role in improving child health. However, many childhood vaccination schedules start at six weeks of age, and many diseases require more than one dose of vaccine to confer adequate protection. As a result, newborns are particularly vulnerable in the time period prior to at least the second dose of vaccine. Young infants are particularly vulnerable to bacterial and viral infections, with mortality highest in the first weeks of life, due to their weak innate immune function [9]. Furthermore vaccines are in development that would protect infants from disease that they may not respond to (respiratory syncytial virus vaccine), and for pathogens with intrapartum transmission (group B streptococcus).

In 1988, the World Health Organization (WHO) estimated that 787,000 newborns worldwide died of tetanus [10]. In response, the WHO called for maternal and neonatal tetanus elimination (MNTE), which is defined as fewer than one neonatal tetanus case per 1,000 live births in all districts per year. Key components of this initiative include routine immunization of pregnant women and women of reproductive age with tetanus toxoid containing vaccine (TTCV), hygienic delivery and cord care practices and strengthening neonatal tetanus surveillance. [10]. The WHO estimated that in 2015, approximately 34,000 newborns died from neonatal tetanus, a 96% reduction from the situation in 1988 [10]. However, as of August 2019, 12 countries have still not attained MNTE status, evoking the need to understand what vaccine implementation efforts in pregnant women are required to resolve underlying bottle necks [11], [12].

In 2012, global recommendations on influenza vaccination stated that pregnant women should have the highest priority for seasonal influenza vaccination in countries considering the initiation or expansion of influenza immunization programs [13]. Despite these recommendations, the introduction of maternal influenza immunization in many LMICs is lagging behind. With new maternal vaccine candidates such as those targeting respiratory syncytial virus (RSV) and group B streptococcus (GBS) in development, there is an urgent need to identify and understand the operational drivers and challenges that may support or hamper high vaccination coverage of new vaccines in pregnant women.

WHO/UNICEF Estimates of National Immunization Coverage (WUENIC) provide annual estimates of immunization coverage of vaccine-preventable diseases (VPDs), including TTCV immunization coverage of pregnant women [14]. TTCV coverage can be assessed using TT2+ (at least two TTCV doses) and protection at birth (PAB) data [15]. PAB is a supplemental method of determining coverage protection (especially where TT2 + is unreliable). To monitor PAB during diphtheria, tetanus, pertussis vaccine first (DTP1) visit, health workers record whether infants were protected at birth by the mother’s TTCV status.

The 10.13039/100004423WHO, supported by the 10.13039/100000865Bill & Melinda Gates Foundation, implemented the Maternal Immunization and Antenatal Care Situational Analysis (MIACSA) project, which started in 2016. The project set out to identify and understand the challenges from, barriers to, and facilitators of existing maternal tetanus immunization services in LMICs as a learning agenda to optimise the pathway for new maternal vaccines. This project aimed to determine how existing health services currently deliver TTCV (and other vaccines) to pregnant women and the attributes associated with effective maternal vaccine service delivery in LMICs. An important goal was to identify what aspects need to be strengthened and what gaps need to be addressed to facilitate the introduction of additional and new maternal vaccines such as influenza, 10.13039/100013560GBS and RSV.

## 2 Methods

### 2.1 Study design and data collection

An abridged methodology is described below. For a detailed overview of the project methodology please refer to the study protocol previously published [16].

**Fig. 1:**
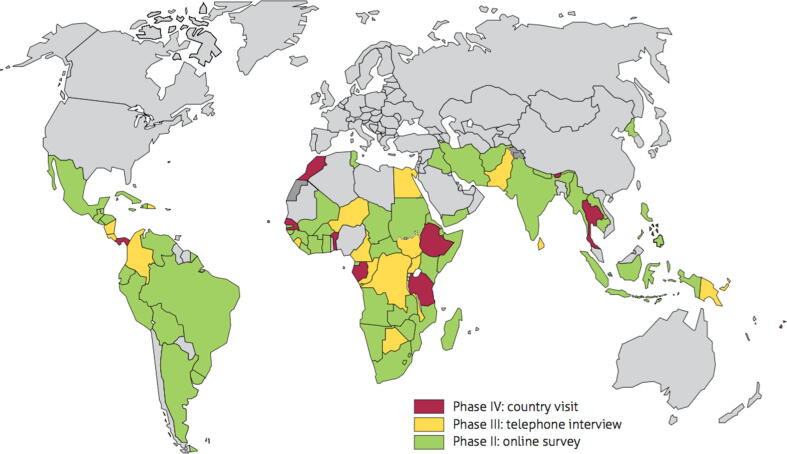
Countries participating in the different study phases.

In summary, between November 2016 and September 2018, a multi-method cross-sectional study was carried out with four phases detailed below Fig. 1.

## 1 Phase I – Published and unpublished data of pre-defined maternal and child health

Published and unpublished data of pre-defined maternal and child health indicators and World Bank Data for economic level in 137 LMICs were reviewed. The classification for the year 2016 was used.

The following databases were used to extract data: Demographic and Health Surveys (DHS)/Multiple Indicator Cluster Surveys (MICS); WUENIC; MNTEdatabase; WHO Maternal, Newborn, Child, and Adolescent Health (MNCAH) policy surveys; World Bank Classification; United Nations (UN) Inter-agency Group for Child Mortality Estimation; trends in maternal mortality: 1990 to 2015, and United Nations, Department of Economic and Social Affairs, Population Division.

## 2 Phase II - global online survey sent to 116 LMICs

An 18 item online survey was developed to collect data on maternal tetanus immunization service delivery models, programme funding, type of disease surveillance, vaccine safety surveillance and maternal vaccines other than TTCV. Non-responders were followed up by telephone and/or email as described in the study protocol [16]. Revisions following queries on missing, erroneous and inconsistent data were carried out through country visits. Service delivery models of interest for maternal tetanus immunization were those within EPI programmes, those within Maternal Newborn Child Health (MNCH) programmes or service delivery models that included a combination of EPI and MNCH. The survey was not sent to the WHO European region because the MNTE initiative is not a priority there.

Following the online survey, countries for Phase III and Phase IV were selected based on performance of maternal immunization as assessed by coverage of TTCV and ANC, geographic representation and recommendations from WHO Regional Offices. The countries were stratified into four groups; high and low maternal tetanus vaccination performance measured as protection at birth (PAB), with a cut-off

of 90%, and high and low ANC performance (with a cut-off of the median ANC4 + coverage in countries with available data). The final country selection was intended to ensure representation from all WHO regions, including high-performing countries, MNTE priority countries (those that had not yet achieved elimination status) and countries with high ANC4 + coverage. If a country was selected and agreed to an ‘in-country’ visit (see below), and the survey for Phase III was not completed prior to the visit, then the survey was administered in-person at the time of the country visit. Selection of countries was not random and health care facilities chosen during ‘in country’ visits were determined by the Ministry of Health of that country.

## 3 Phase III - telephone and face-to-face interviews for 26 LMICs

A 91-item survey with more detailed questions on the domains included in the online survey was developed by the MIACSA Expert Advisory Panel. The questionnaire was first pilot tested in two countries, and adapted based on the comments provided. In-depth telephone and in-person interviews were conducted with EPI and MNCH programme officers in a selection of countries [16]. Twenty-six countries participated in the telephone survey, namely Benin, Bhutan, Botswana, Cameroon, Democratic Republic of the Congo (DRC), Republic of Congo, Colombia, Costa Rica, Dominica, Egypt, Ethiopia, Fiji, Gabon, Malawi, Morocco, Nicaragua, Niger, Pakistan, Papua New Guinea, Senegal, Sierra Leone, South Sudan, Sri Lanka, Thailand, Tanzania and Uganda.

## 4 Phase IV-In-country visits for 10 LMICs

In-country visits were conducted in ten selected countries. Week-long, in-country visits took place in Benin, Bhutan, Ethiopia, Fiji, Gabon, Morocco, Panama, Senegal, Tanzania and Thailand. Data collected included in-depth key informant interviews, focus group discussions, and observations of clinical practice and review of available documents at health care facilities.

### 5 Statistical analysis

All data were imported into Stata (release 15, StataCorp LCC, Texas) for analyses. Data were checked for completeness and consistency. Variables collected using a multiple-answer option were split into individual variables reflecting each answer category.

PAB was used as dichotomous variable: low: PAB < 90% vs. high: PAB>=90%. ANC performance was defined as the proportion of pregnant women who attended one ANC visit during their last pregnancy (ANC1) and the proportion of pregnant women who attended four or more ANC visits during their last pregnancy (ANC4 + ). To measure EPI performance, data from the desk review (phase I described above) on coverage of the third dose of the DTP vaccine (DTP3) and the third dose of the pentavalent vaccine (Penta3) was used as a proxy.

Summary measures (proportions, means, medians and standard errors) were calculated for all variables using univariate analysis. To identify maternal tetanus immunization service delivery models and process components favourable to the vaccination of pregnant women, associations of different service delivery process components with PAB coverage and with country groups were established. Bivariate analyses were conducted to assess the relationship between different variables according to specific hypotheses. The significance of the relationship was tested with Fisher’s exact test. A two- sided *P-*value of 0.05 or below was considered as significant throughout.

Generalized estimating equations (GEE) models were used to calculate p-values for group-differences at the health facility level, taking into account the correlation structure of the sample of 96 health facilities in 10 countries.

Countries were grouped (1) according to high and low PAB coverage, and (2) according to a latent variable estimating the capacity of the health system as regards the protection from vaccine preventable diseases. To create this latent variable latent class analysis (LCA) was used. LCA enables the characterization of an unobserved (latent) variable through analysis of the structure of the relationship among several observed variables [17]. LCA therefore allowed multiple indicators to simultaneously contribute to the definition of a categorical latent variable, here by including several MNCH and EPI performance indicators into the LCA model. The countries were then assigned to the category with the highest probability according to the model. The variables included in the LCA model were PAB, TT2+, DPT3, ANC1, ANC4+, neonatal mortality rate and maternal mortality ratio. The LCA generated four country groups and a hypothesized gradient in the level of protection provided against vaccine preventable diseases (VPDs) to mothers and infants was confirmed for MNTE status. The four groups are defined as:

Group 1: Currently ***very limited potential*** (VLimP) to protect mothers and their infants from VPDs (limited ANC and EPI performance)
Group 2: ***Limited potential*** (LimP) to protect mothers and their infants from VPDs (moderate ANC and EPI performance)
Group 3: ***Moderate potential*** (ModP) to protect of mothers and their infants from VPDs (mostly successful ANC and EPI performance)
Group 4: ***High potential*** (HighP) for protection of mothers and their infants from VPDs (successful ANC and EPI performance)

## 6 Results

The global online survey was answered by 97/116 (84%) countries, of which two provided incomplete responses. These were excluded from the analysis. Twenty-six countries completed Phase III (telephone and/or face-to-face interviews). In each of the country visits, between 6 and 14 health facilities selected by the Ministry of Health, were visited leading to a total of 96 health facility visits and health facility programme officer interviews across countries.

### 6.1 Maternal tetanus immunization policy and targets

From the 95 countries responding to the online questionnaire, 84/95 (88%) reported having a written national policy on vaccinating pregnant women against tetanus. Four countries reported that related guidelines were included in another health policy (Equatorial Guinea, Mali, Nepal and Togo). Overall, 88/95 (93%) of the countries had either a policy or guideline for maternal immunization.

**Fig. 2:**
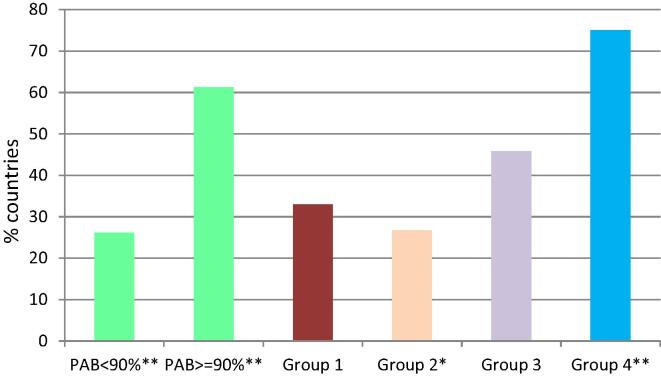
Countries with maternal tetanus immunization targets of 90% and over. Data from online survey (n = 88) P - v a l u e s * : < 0 . 05; **: < 0 . 01 ; Group 1: very limited potential (VLimP); Group 2: limited potential (LimP); Group 3: moderate potential (ModP); Group 4: high potential (HighP); PAB: protection at birth.

Information about maternal tetanus immunization targets was available from the online survey, where 95 countries reported their national targets, and the frequency of reporting. Of the 88 countries with a policy or guideline, 39 (44%) had a target > 90%. Seven countries (8%) did not indicate what the national target was, or indicated a target < 25%. To document whether targets were set based on the current national coverage rates, or based on ideal rates, the targets were compared between countries with high and low PAB coverage. Countries with high PAB coverage significantly more often reported targets of at least 90% (*P*-value 0.002). Group 4 countries (HighP) were also more likely to have targets of at least 90% (*P*-value 0.001), whereas Group 2 countries (LimP) were less likely to have these targets (*P*-value 0.023). On the frequency of reporting, countries mostly reported coverage annually (49%), while 17% reported monthly (Fig. 2).

The country visits demonstrated that national maternal tetanus immunization targets were not necessarily available or known at the health facility level. During the health facility visits, 80/96 (83%) health facility managers reported having maternal tetanus immunization targets for their health facility. At 14 heath facility visits, staff were unaware of national targets. These 14 facilities were located across six countries.

### 6.2 Service delivery models for maternal tetanus immunization

To compare different service delivery strategies used in countries, the organization of service delivery process components was examined, including procurement, programme planning and management, vaccine distribution, training and supervision and vaccine administration to pregnant women. In nearly all countries, EPI was responsible for procurement (88/95; 93%) and distribution (86/95; 91%) of TTCV. For planning and management responsibilities, this was often shared between EPI and ANC (39/95 52%) and in 49/95 (52%) countries planning and management was the responsibility of EPI only. (Table 1). No strategy emerged as superior with regard to maternal tetanus immunization performance as measured by PAB.

**Table 1:** Maternal tetanus immunization service delivery responsibilities at the national level.

	Procurement	Planning and management	Distribution	Training and supervision
Expanded Programme on Immunization (EPI) only	88	49	86	69
Antenatal care (ANC) only	0	6	0	3
EPI + ANC	2	39	5	23
Other*	5	–	2	–
Unsure	–	1	2	–

* Includes other government entities or international non-governmental organizations

**Fig. 3:**
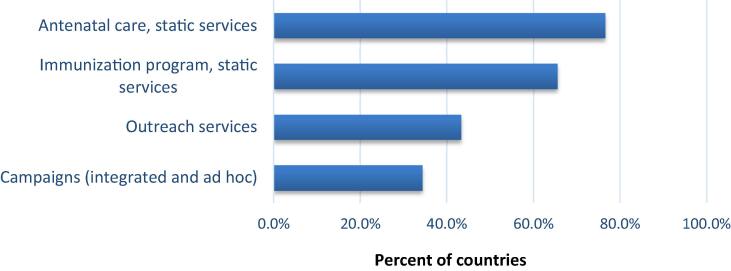
Service types offering routine maternal tetanus immunization. *Data from online survey among 95 countries (five missing answers)*.

Respondents were asked to indicate the estimated proportion of routine maternal TTCV administered at each service delivery type, such as facility-based ANC, facility-based EPI/immunization clinic, outreach services or integrated campaigns. Therefore, more than one answer could apply. Countries were found to use a mix of services to administer maternal TTCV, which were often dependent on the local setting, with 77% administered in ANC clinics, followed by 66% in EPI clinics (Fig. 3). The countries that reported outreach to be the most common setting for maternal tetanus immunization included Bangladesh, Lao People’s Democratic Republic, Myanmar, and Sudan.

More group 1 (VLimP) countries vaccinated the majority (>50%) of pregnant women at EPI/immunization services in health facilities, compared to other countries (P-value 0.029). Group 2 (LimP) countries were less likely to vaccinate pregnant women in EPI clinics (P-value 0.019) but more often reported vaccinating pregnant women primarily through outreach activities compared to the other countries (P-value 0.009). Group 4 (HighP) countries, more often reported vaccinating over 90% of pregnant women at facility-based ANC services compared to the other countries (P-value 0.047).

Associations were observed between organization at the national level and services more likely to deliver TTCV to pregnant women. From the online survey, in 22/29 (76%) countries where EPI services provided maternal tetanus immunization to > 50% of all pregnant women, the EPI was responsible for planning, management, training and supervision. Countries providing vaccinations through ANC services to > 50% of the women more often shared management responsibilities between EPI and MNCH programmes (29/43, 65%).

The proportion of women receiving TTCV at the same time as their ANC appointment was lowest in Group 1 countries and highest in Group 4 countries. When > 75% of women received TTCV and ANC in a single visit, PAB > 90% was more likely (*P*–value 0.032). This association remained unchanged when controlling for country income level. A one-stop approach was less common in Group 1 countries (*P*-value 0.001). However not all better-performing countries used a one-stop approach. In four of the Group 4 countries, <25% of pregnant women received the TTCV at the same time they received ANC.

### 6.3 Human resources

The level of skilled staff trained to vaccinate pregnant women in ANC services was explored in the 26 telephone interviews. In most countries, registered nurses were the most frequent skilled staff vaccinating, followed by enrolled or auxiliary nurses. The proportion of countries reporting medical doctors providing vaccines increased at tertiary level facilities, with clinical officers most often listed at the secondary level and registered nurses at the primary level. Capacity to provide maternal tetanus immunization services (including adequate space, cold chain, vaccine supply, skilled staff) at facilities currently delivering ANC was not found to differ between countries with low and high PAB coverage.

During the 10 country visits, health facility managers were asked about skilled staff administering maternal vaccines at their facility. Of the 96 health facility interviews 76/96 (79%) were at primary level where vaccines were most often provided by registered nurses or midwives. The remaining 20/96 (21%) were at secondary or tertiary level facilities. In Group 4 (HighP) countries more registered nurses or midwives provided vaccination to pregnant women (P value 0.032) compared with other groups while Group 2 and Group 3 countries more often relied on enrolled nurses or auxiliary nurses.

### 6.4 Record keeping

**Fig. 4:**
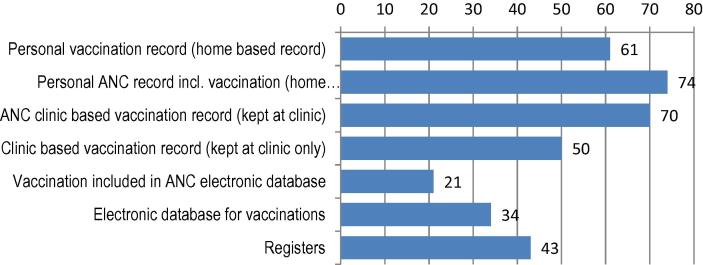
Countries with personal-held record keeping of past immunization. *Online survey in 95 countries (two missing answers)*.

Various approaches to maintaining maternal immunization records existed (often concomitantly) in the 95 countries that participated in the online survey. These included personal ANC records and/or vaccination cards held by the pregnant woman (any home-based record: 85/92; 92%), or clinic-based records (any clinic-based information: 83/92; 90%) (Fig. 4). Electronic information systems existed in 40/92 (43%) countries. Of these, 34/92 (37%) were for vaccinations and 21/92 (23%). for ANC. Electronic information systems were more often reported in Group 4 (HighP) countries (P-value 0.024). In some countries, vaccination details were simultaneously registered in several places, within multiple, coexisting documentation formats.

Information from EPI and MNCH country programme officers obtained from the telephone interviews further revealed that many countries relied on maternal recall (16/26; 62%) if no written documentation on immunization details was available. Group 1 (VLimP) countries relied on maternal recall more often than other groups (P-value 0.014), and Group 4 (HighP) less as compared to other countries (P-value 0.046).

During the country visits, 73% of health facility managers confirmed that, in principle, records could be linked between the mother and the child. Modalities included combined mother–child health or vaccination booklets, mother-held vaccination cards, or linked ANC and child registries. In practice, however, there were inconsistencies in completeness of the documented record linkage of immunization details.

### 6.5 Immunization safety surveillance

The majority, 81/95 (85%), of countries which participated in the online survey had established an immunization safety surveillance system, which allows the identification of adverse events following immunization (AEFI).

During the country visits, such a safety surveillance system was available in 70/90 health facilities (78%, six missing responses). Of the 70 facilities reporting an AEFI system, only 11% distinguished between pregnant and non-pregnant women. The proportion was slightly (and significantly) higher in the high performing countries, but still remained below 20%.

### 6.6 Delivery of vaccine and cold-chain

In the telephone interviews with country programme EPI and MNCH officers, respondents were asked how they perceived the quality of the transportation and storage conditions of vaccines in their country at national, regional and health facility level. There were no differences in the perceived quality of transport and storage of vaccines between the country groups, and satisfaction with the processes did not differ between countries with high and low PAB coverage. Countries with high PAB coverage more often stated that they were very satisfied with the process of transporting vaccines from health care institutions to vaccination centers, as compared to countries with low PAB, which reported to be satisfied, but to a lesser degree (data not shown).

The capacity of the storage system was further explored during the country visits to 96 health facilities (Table 2). Only four health facilities from two countries had concerns with their on-site cold chain capacity or with vaccine supply. These problems were reported in Group 2 countries only (LimP) (P-value 0.046) who were also more likely to report TTCV stock outs. Stock-outs of TTCV in the past were reported in 17 health facilities across six out of ten countries.

**Table 2:** Cold chain capacity, interviews with 96 health facility managers in 10 countries.

Does your facility have onsite cold chain capacity for vaccine storage?							
	PAB < 90%	PAB ≥ 90%	Group 1	Group 2	Group 3	Group 4	Total
No n (%)	3 (6.38)	1 (2.4)	–	4 (8.9)	0 (0)	0 (0)	4 (4.2)
Yes n (%)	44 (93.62)	41 (97.6)	–	41 (91.1)	33 (1 0 0)	17 (1 0 0)	91 (95.8)
Total	47 (1 0 0)	42 (1 0 0)	–	44 (1 0 0)	33 (1 0 0)	17 (1 0 0)	95 (1 0 0)
Fisher's exact		0.62		**0.046**/0.009*	0.293	1	
**Does your facility have a sufficient supply of TTCV?**							
	**PAB < 90%**	**PAB ≥ 90%**	**Group 1**	**Group 2**	**Group 4**	**Group 3**	**Total**
No n (%)	2 (4.35)	6 (14)	–	6 (13.6)	2 (6.06)	0 (0)	8 (9)
Yes n (%)	44 (95.65)	37 (86)	–	38 (86.4)	31 (93.94)	18 (1 0 0)	87 (91)
Total	46 (1 0 0)	43 (1 0 0)	–	44 (1 0 0)	33 (1 0 0)	18 (1 0 0)	95 (1 0 0)
Fisher's exact		0.149		0.139	0.710	0.345	
**Have you experienced any stock outs of TTCV at your facility?**							
	**PAB < 90%**	**PAB ≥ 90%**	**Group 1**	**Group 2**	**Group 4**	**Group 3**	**Total**
No n (%)	41 (89.1)	31 (72.1)	–	32 (72.7)	29 (87.88)	17 (94.44)	78 (82.1)
Yes n (%)	5 (10.9)	12 (27.9)	–	12 (27.3)	4 (12.12)	1 (5.56)	17 (17.9)
Total	46 (1 0 0)	43 (1 0 0)	–	44 (1 0 0)	33 (1 0 0)	18 (1 0 0)	95 (1 0 0)
Fisher's exact		0.059/0.131*		**0.033/**0.133*	0.401	0.180/0.092*	

Group 1: *very limited potential* (VLimP)

Group 2: *Limited potential* (LimP)

Group 3: *Moderate potential* (ModP)

Group 4: *High potential* (HighP)

* p-value from generalized estimating equations

**Fig. 5:**
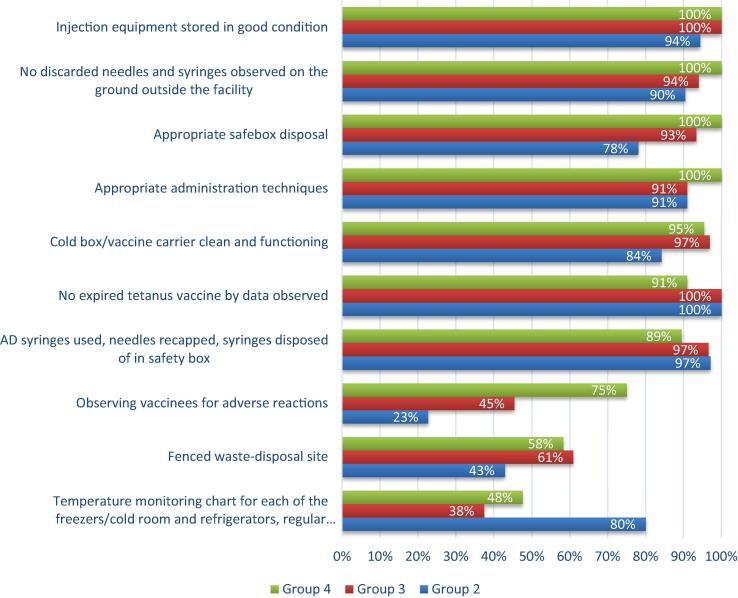
Vaccine storage, administration and waste disposal practices observed during health facility visits (n = 95) in ten countries. No Group 1 countries were included in the country visitsGroup 2: *Limited potential* (LimP)Group 3: *Moderate potential* (ModP)Group 4: *High potential* (HighP)

Differences were observed among health facilities during the 10 country visits in the management of vaccines, the administration of vaccines, and waste-disposal (Fig. 5). Group 2 country (LimP) health facilities were less likely to have functional vaccine carriers (P-value 0.061) and less likely to have adequate safety disposal boxes (P-value 0.02).

### 6.7 Other vaccines

From the desk review, 58/98 (59%) countries with available data had influenza vaccine included for pregnant women in their routine immunization schedule. No data on other maternal vaccines was available for 39 LMICs from the desk review database. However, maternal influenza immunization was not offered to pregnant women on a routine basis in any of the 58 countries. At the same time, responses to the MIACSA online survey indicated that 24/95 (25%) had introduced maternal influenza immunization and 9/95 (9%) maternal pertussis immunization. Most of the countries with maternal immunizations other than TTCV were located in Latin America. The responsibility for procurement, planning and management of maternal vaccines other than TTCV was not always with the same organizational entity as for the maternal tetanus programme. When this was observed during the in-country visits, coverage was found to be significantly higher for maternal tetanus immunization than for the additional maternal vaccine (influenza).

## 7 Discussion

The findings from the MIACSA project shed light on how existing health services in countries currently deliver TTCV (and other vaccines) to pregnant women and on attributes associated with effective maternal vaccine service delivery in LMICs. It also reveals bottlenecks and opportunities to optimize delivery of maternal vaccines that may be required for the introduction of additional maternal vaccines in the future. Successful approaches identified in this project include target setting, integrated programme coordination between EPI and MNCH and operational approaches that maximise a woman’s access to maternal immunization at the time of ANC.

In terms of policy and targets for maternal immunization, the majority of countries (>90%) had a maternal tetanus immunization policy in place, which is to be expected given that MNTE efforts have been ongoing for over two decades [12]. Only half of these policies, however, include TTCV coverage targets > 90%, and these targets are not always known at the health facility level. Along with national recommendations, setting ambitious, but, attainable country-specific targets is recommended by the WHO as part of the Global Vaccine Action Plan [18], which calls on countries to meet coverage targets in every region, country and community by 2020 [18]. The MIACSA results confirm that increased use of targets are more frequent in group 4 (HighP) countries. Setting pre-defined targets is not only important to measure success following implementation, but may also improve coverage by incentivising vaccinators to achieve the target and/or identify shortcomings leading to further interventions to address these. Therefore, continued awareness and reinforcement about global targets at a country level should allow countries to better monitor, evaluate and optimize their programmes.

With regards to service delivery strategies for TTCV, the MIACSA project provides the most detailed typology of maternal tetanus immunization service delivery strategies in LMICs to date. It found good collaboration between EPI and MNCH at various stages of service delivery in most, and particularly in group 4 (HighP) countries. In nearly all countries, procurement and vaccine distribution are managed primarily by immunization programmes, which appears logical due to the expected economies of scale and logistical synergies arising from joint procurement and storage of vaccines. Other areas, such as planning and managing of maternal immunization programmes and staff training were more often coordinated jointly by national EPI and MNCH programmes, particularly in countries where the administration of vaccines to pregnant women is primarily done in ANC facilities. Interestingly, countries with very limited or limited potential (group 1 and 2) were more likely to rely primarily on services other than ANC services, i.e. EPI clinics or EPI outreach activities. Whether factors affecting these strategy choices are driven by limitations of ANC service capacity or other factors still needs to be better understood.

Integrated service delivery approaches with EPI or MNCH programmes taking the lead in areas related to their expertise appears to add value. The MIACSA project found a correlation between high performance (PAB > 90%) and receipt of TTCV at the same time as the woman’s ANC visit. This association remained when country income level was controlled for. Building on this finding and supporting close collaboration between EPI and MNCH/ANC services including enabling maternal vaccination to take place simultaneously with ANC visits may hold the key to identifying and addressing shortcomings with the expansion of maternal immunization programmes.. However, this needs to be context specific as building vaccination into ANC may not be the best approach if ANC coverage is low.

Workforce shortcomings remain a challenge to increasing opportunities for vaccinating pregnant women. Such challenges were observed during MIACSA country visits, in particular at decentralized levels, as have been reported in the literature [19]. With regards to responsibilities for administering vaccines to pregnant women at health facility level, the small sample of health facilities visited limits generalizable conclusions. However, it is worth mentioning that at primary level facilities often, there was only one health worker performing the job of ANC service provider and maternal vaccinator. This “integration” of roles may work well currently but may also present challenges if workload of any individual health worker is increased by the addition of further maternal vaccines in the future. In addition, the increased involvement of nurses observed in primary level health facilities compared to doctors in secondary and tertiary facilities is worth further exploration to understand how this distribution of responsibility may impact on the introduction of additional maternal immunizations. Here, establishing multi-disciplinary health workforce teams may help to overcome shortcomings and improve efficiency of services.

Accurate record keeping of personal immunization details enables vaccinators to confirm the vaccination status of pregnant women. The MIACSA project found that both home-based and facility-based records are used in a large majority of countries. In particular, home-based records appeared valuable to track women’s immunization status across pregnancies and their migration across different health facilities. The challenges observed with paper-based record keeping found during country visits were similar to those previously described in literature, such as poor data quality, limited timeliness of information for reporting purposes and time intensive for staff [20]. Often, duplication of paper-based recording was evident and electronic record keeping not in place. Furthermore, maternal recall was relied upon over 60% of the time to inform past maternal immunization history and health facility managers reported that although linkage of maternal and child records was possible, this occurred inconsistently. A systematic review on the validity of vaccination cards and parental recall to estimate vaccination coverage has reported poor concordance, with maternal recall reported to both overestimate and underestimate coverage [21]. Although the sample size of health facilities visited is small, and not necessarily representative, these findings, taken together, suggest that further strengthening of recording systems is warranted.

A report by the WHO Strategic Advisory Group of Experts (SAGE) on Immunization working group on quality and use of immunization and surveillance data found that despite considerable guidance documents available globally, there was a gap in materials identified for life course and special populations such as pregnant women [22]. Electronic information systems have been suggested as a way to improve data completeness, timeliness and data integrity [23]. However, this is highly dependent on the function and design as well as the completeness and accuracy of data contained [24]. For maternal immunization programmes, utilising electronic information systems may improve data collection and allow access to data (e.g. in case of home-based record loss) but overall success will be dependent on the local health system readiness. Improving data use in resource limited settings requires identification of a well-defined problem to be addressed, infrastructure (e.g. reliable internet access and stable electricity), sustainable financing, health worker training and feedback mechanisms on data generated [18], [25].

Along with reporting of vaccination in pregnancy, immunization safety surveillance systems are an important component of a maternal immunization programme, given the unique safety concerns in this population [26]. Having a safety surveillance system that is able to identify a pregnant woman as the recipient of the vaccine may facilitate early detection of any safety concerns among this population and/or provide data to maintain confidence in the safety of the programme. Across the large majority of visited countries, MIACSA found that safety-reporting forms did not differentiate AEFI in pregnant and non-pregnant women. Although WHO recommendations and tools for reporting of AEFI in pregnant women exist [27], few countries are yet to implement them fully [28]. In addition, qualitative data from the health facility visits undertaken in this project highlight the need for on-going education and training of health workers given uncertainty among some health facility managers about the nature and frequency of AEFI in pregnant women. Recommendations arising from this project include for LMICs to adjust their current AEFI reporting to be able to differentiate pregnant women and to increase education of health workers to be able to recognise and report AEFI.

The MIACSA project was primarily focused on maternal tetanus immunization. However, some study findings relate to the use of other vaccines targeting pregnant women. Countries that had introduced influenza or pertussis vaccines into their maternal immunization schedule were significantly more likely to have a PAB coverage >=90%. This may indicate that countries with a well-functioning maternal tetanus immunization programme are able to expand vaccination of pregnant women to include additional vaccines. In two of the visited countries, influenza vaccine had recently been introduced but had considerably lower coverage rates compared to TTCV. Being limited to service delivery aspects, MIACSA was not powered to identify factors contributing to different coverage levels. However, it was striking that for both vaccines, different service delivery logistics were in place. In these two countries, influenza vaccine was managed outside of services providing TTCV to pregnant women. This meant that pregnant women had to seek influenza vaccine from clinics at different locations. Accordingly, a recommendation from the observations of the MIACSA project to prevent low uptake in pregnant women would be to allocate responsibility for delivering new vaccines into existing maternal immunization services or at least to ensure robust referral systems that minimise the risk of drop out of pregnant women between services.

This study has several limitations. The different data sources used in the MIACSA project meant that inconsistencies between the different databases were observed for some quantitative and policy-related indicators. This may have been due to the different time-points the information was collected, inconsistent definitions across data sources, or different sampling methodologies. Besides different sources of data, inaccurate reporting cannot be fully excluded despite efforts made by the countries to validate the information provided to the study team. Another limitation was the relatively small number of countries visited, and of health facilities visited within a given country. Health facilities visited were not randomly selected but were selected by the local Ministries of Health. They are not therefore necessarily representative of the entire country but rather may reflect a likely higher-performing part of the health system within a particular country. It needs to be acknowledged that although Phase II through to Phase IV of the MIACSA project provide useful insights into how maternal tetanus immunization is currently delivered to pregnant women in LMICs, the ten countries visited are not representative of all LMICs. The findings therefore need to be interpreted with this in mind.

A strength of the MIACSA project however was related to how the four phases were conducted in sequence, each building on the next. Each subsequent phase added a level of granularity to the data collected for each of the areas of interest. In addition, the mixed methods approach allowed for quantitative and qualitative data collection along with direct observation at a healthcare facility level during the in-country visits. This added depth and richness in understanding and interpretation of the data which would not have been possible if the study was limited to only one phase or methodology. It also facilitated regional balance, representation of different vaccine delivery models and inclusion of multiple performance category countries.

## 8 Conclusion

Specific recommendations arising from the MIACSA project in relation to strengthening existing services currently delivering maternal tetanus immunization in LMICs are as follows: ;

In each of the areas listed, countries should

• **Policy:** Establish and maintain vaccination targets for pregnant women and monitor progress at regular intervals.
• **Service delivery:** Define clear responsibilities and ensure functional collaborations between EPI and MNCH from national to health facility level. Promote efficient care practice approaches such as vaccination at the same time as ANC visits.
• **Human resources:** Invest in strengthening the health workforce both in numbers and in capacity to safely and effectively provide maternal immunization specifically addressing shortages that may be evident in the context of new vaccine introduction. This may include establishing multi-disciplinary health workforce teams, to help overcome human resource shortages and support demand creation.
• **Record keeping:** Improve the design and use of existing records to reduce the reliance on maternal recall to establish vaccination status. Consider use of electronic record approaches where paper-based recording of vaccination poses challenges in terms of collection, data quality and/or data analysis, but only after an assessment of capabilities (including internet access, reliable power supply, trained staff) and availability of sustainable funding.
• **Cold chain:** Assess capacities of their cold chain system before introducing a new maternal vaccine.
• **Immunization safety surveillance:** Adjust current AEFI reporting to be able to differentiate pregnant women and increase education of health workers to be able to recognise and report AEFI appropriately.
• **Introduction of other maternal vaccines:** Aim to ensure that the service delivery of new vaccines is integrated with existing delivery channels for TTCV, where these achieve high coverage. Where TTCV coverage is low, the introduction of new maternal vaccines could provide important opportunities for countries to review and strengthen the service delivery of both TTCV and ANC services.

While maternal tetanus immunization is operational in most LMICs introducing additional maternal vaccines presents many challenges. It is hoped that the findings from the MIACSA project and the implementation of these evidence-based recommendations will contribute to the achievement of global MNTE and contribute to a robust platform for the introduction of new maternal vaccines in the future.

## Declaration of Competing Interest

The authors declare that they have no known competing financial interests or personal relationships that could have appeared to influence the work reported in this paper. The authors alone are responsible for the views expressed in this article and they do not necessarily represent the views, decisions or policies of the institutions with which they are affiliated.
